# Educational resources used by medical students in primary healthcare rotation: A cross sectional study

**DOI:** 10.12669/pjms.322.9784

**Published:** 2016

**Authors:** Ali Al-Hazmi

**Affiliations:** Dr. Ali Al-Hazmi, MBBS, FFCM. Department of Family and Community Medicine, College of Medicine King Saud University, Riyadh, Saudi Arabia

**Keywords:** Educational resources, Primary Health Care, Medical Students

## Abstract

**Objective:**

To identify what educational resources are used by medical students for their personal study during Primary health care (PHC) clinical rotation and the reasons for making these choices at King Saud University in Riyadh, Saudi Arabia.

**Methods:**

A survey of 176 male and female medical students was conducted during PHC rotation. A self-administered questionnaire ascertained the type of educational source with reason and preferred type of teaching method. Responses by male and female students were compared by using Pearson’s Chi-square tests.

**Results:**

Of the 176 students, 85.8% used handouts, 77.3% used the internet, and 46.6% used textbooks. Of the three types of resources, 14.8% used one, 31.8% used all three sources, and 53.4% used two sources. Reasons for selecting a resource were; educational materials are up to the point (88.6%), convenient (85.2%), reliable (77.3%) fit the learning style (77.3%), exam focus (60.8%), recommended by seniors (57.4%), recommended by department (56.8%). The preferred teaching method was lecture (79.5%), and least preferred was student presentations (55.1%). Female medical students used internet related material greater than the males (86.9% versus 68.5%; p value <0.001), and tended to utilize more than one educational resource than male students.

**Conclusion:**

Medical students used multiple resources for relevance and convenience. Female students used network resources more than male students.

## INTRODUCTION

Medical education faces challenges in teaching and learning methods for medical students. Such a challenge is exponentially growing given the rapidly advancing knowledge and at the same time increasing the number of available resources to medical students. It is reported that time taken for development of equivalent new knowledge was 50 years in 1950 which was reduced to 3.5 years in 2010, and is projected to be 73 days in 2030.^1^ It is imperative to understand the processes through which medical students develop learning experiences to embrace desired competencies.^2^ Cognitive learning theory emphasizes that quality of new knowledge be based on activation of prior or existing knowledge; whereas experiential learning theory is based on that effective learning occurs through gaining experience; a cyclical process linking concrete experience with abstract conceptualization.^3^ Medical students may have individual learning styles, and such styles of learning may differ by several factors like age, sex, and year of study. Approaches to learning from preclinical and clinical years differ as they move from conceptual learning to deeper types of learning styles.^4^,^5^

At present medical students are using a variety of sources to learn that are made available to them through designed curriculum and their institutions. Learning experiences are essential in primary healthcare clinical rotations as most of the practice is done at primary healthcare level compared to advanced healthcare disease spectrum of admitted patients.^1^ Primary health care rotations are one of the important rotations where medical students integrate their core knowledge supplementing it with sequential learning by relating it to several domains. Therefore, use of educational resources may potentially differ from primary health care rotation to another; some were using more web-based resources and some using traditional sources like textbooks.^6^ Medical institutions and faculty strive to address challenges by acquiring evidence to develop structured curriculum for students in primary health care for intended learning outcomes. Hence, it is necessary to determine the students’ practices to accomplish their learning goals. King Saud University is one of the oldest and largest institutions in Riyadh Saudi Arabia where we designed this study to identify the type of educational resources and reasons for its selection during primary healthcare attachment by medical students at College of Medicine in Riyadh Saudi Arabia.

## METHODS

The author performed a descriptive cross sectional study among 228 male and female medical students during their 4th-year primary health care rotation, at the College of Medicine of King Saud University. The study was carried out between August 2014 and May 2015. The author took the students informed consent to participate in the study. As part of undergraduate academic curriculum male and female groups separately rotated in male and female primary healthcare clinics. All students were invited to participate in this study. A self-administered questionnaire was distributed to consenting students to ascertain educational resources and reasons to use these resources, along with preferred teaching method. About 176 (77.2%) of the students had responded to this study. The questionnaire was designed by the teaching faculty using some questions from reported studies.^6^ The survey tool comprised of three main sections. The first section was concerned with types of education resources on which students relied to learn during primary healthcare rotation, like handouts by instructors, use of conventional textbooks on primary health care, use of electronic methods of medical education including websites and phone apps. The second section comprised of the type of teaching method favored by students to achieve their educational learning objectives that included lectures, discussion on case presentations; clinical attachments, student led seminars, and student presentations. The third part was related to the factors that determined the selection of educational resource.

Data analysis was performed on the statistical software of SPSS 21. Descriptive statistics (frequencies and percentages) were used to quantify the responses of educational resources, type of preferred teaching method, and reasons for selecting the educational source by males and female students. Pearson’s Chi-square test was used to compare the responses of male and female students. A p- value of less than 0.05 was considered to report the statistical significance of the results.

Ethical approval for the study was obtained from King Saud University Institutional Review Board. Confidentiality was maintained by assigning a unique identity number to each questionnaire. No incentives were given to study participants.

## RESULTS

Of the 176 students, 92 (52.3%) were males, and 84 (47.7%) were females. Both male and female students in primary health care rotation used multiple and diverse educational resources. The majority of them used handouts (85.8%), and internet based resources (77.3%), and less than half of them used textbooks (46.6%). (Table-I). Among these fourth year, medical students females significantly used more internet based educational resources than male students did (86.9% versus 68.5%; p value <0.003). Multiple sources like handouts, web-based, or textbooks, when counted by number, females were about twice more likely to use more than one resource than males (Table-I).

**Table-I:** Distribution and comparison of responses towards use of education sources and number of sources between male and female Students.

Education source	Male (n=92)		Females (n=84)		Total (n=176)		Chi square	P - value

	No	%	No	%	No	%		
Handout	77	83.7	74	88.1	151	85.8	0.697	0.4037
Internet	63	68.5	73	86.9	136	77.3	8.489	0.0036
Books	43	46.7	39	46.4	82	46.6	0.002	0.9671
Number of sources								
One	17	18.5	9	10.7	26	14.8	4.958	0.0838
Two	52	56.5	42	50.0	94	53.4		
Three	23	25.0	33	39.3	56	31.8		

Males and females differed by the type and number of educational resources used, however, they did not differ by the reason for using any particular resource (Table-II). More than 80% of males and females used the particular educational resource for it being “to the point” meaning relevant, suitable, and focused and that the resource was convenient to use. More than 70% of males and females used the resource for the reason of it being reliable and was according to their learning style. (Table-II). About 60% or a little less than that number of students used resources for examination focus, recommended by the senior students or recommended by the department. (Table-II)

**Table-II:** Distribution and comparison of responses towards the d factors that determine the selection of education resources between male and female students.

Factors	Males (n=92)		Females (n=84)		Total (n=176)		Chi square	P-value

	No	%	No	%	No	%		
To the point	78	84.8	78	92.9	156	88.6	2.842	0.091
Convenient	78	84.8	72	85.7	150	85.2	0.030	0.861
Reliable	69	75.0	67	79.8	136	77.3	0.567	0.451
Fit the learning style	71	77.2	65	77.4	136	77.3	0.001	0.973
Exam Focus	57	62.0	50	59.5	107	60.8	0.109	0.741
Recommended by senior students	55	59.8	46	54.8	101	57.4	0.453	0.501
Recommended by the Department	53	57.6	47	56.0	100	56.8	0.049	0.824

Reported teaching methods that helped these students to achieve their objectives were lectures (79.5%), case presentations (72.7%), clinical attachment (64.8%), student led seminar (60.2%), and student presentations (55.1%). Male and female students did not differ by these areas, only that females tended to use lectures more than males (83.7% versus 75%) but this difference was not statistically significant. (Table-III).

**Table-III:** Distribution and comparison of responses towards the teaching methods that helped in achieving the educational objective between male and female students.

Teaching methods	Males (n=92)		Females (n=84)		Total (n=176)		Chi square	P - value

	No	%	No	%	No	%		
Lecture	77	83.7	63	75.0	140	79.5	2.041	0.153
Case presentation discussion	68	73.9	60	71.4	128	72.7	0.137	0.711
Clinical attachment	57	62.0	57	67.9	114	64.8	0.670	0.413
Student led seminar	58	63.0	48	57.1	106	60.2	0.638	0.424
Student presentation	48	52.2	49	58.3	97	55.1	0.673	0.411

## DISCUSSION

This study showed that handouts were the prime source of educational resource followed by internet based resources and lesser use of textbooks. At the same time lectures without handouts may result in a negative effect on student learning.^7^ In contrast to study from UK that reported use of textbooks as the main educational resource in primary health care rotation,^6^ this study showed that students use handouts from lectures followed by internet use. Blended approach (internet based and lectures) have been reported to enhance students learning.^8^ This study setting was for fourth year medical students and perhaps they were in the process of evolving their self-directed styles of learning.^9^ The students emphasized use of to the point, relevant and focused resources and methods to achieve their educational objectives,^5^ not mainly putting focus on examination based learning.^6^

This study showed a fine difference between males and females students on using the type of educational resources, the females used more web-based resources compared to the males. There are several reports in literature that have shown differences in medical students; better learning and performance among females.^10^,^11^ However, later some insight is provided in this difference to be gained by male students as being more confident in clinical practice whereas females being more accurate.^12^,^13^ Moreover a study from India reported that female student’s self-assessment was better than male students making them more objective in focused learning.^11^ Likewise, a study from Saudi Arabia showed a difference in learning style of male and female students, but it did not have an impact on achievements or grade point average.^10^ The primary health care rotation is one of the crucial rotations for medical students and in many counties such as England where 13% of the curriculum is devoted to it.^14^ Hence it is essential to comprehend preferred teaching and learning methods by students during this rotation.

Strength of this study is that in this setting male and female students are taught separately and uniformity of responses indirectly reflect standardized teaching and learning resources and methods. Secondly, a large number of students participated, and completed the questionnaires without missing values. Of the 99 female students 84 participated, and of the 150 male students 92 participated. Overall response was more than 70%, if there was any selection bias then it was minimal as majority of results did differ between males and females. By design, students’ perceptions were obtained as a snapshot and relevant interventions would improve learning techniques. There could be one limitation that reported use of resources and reason for using learning and teaching methods were subjective and may change by subject topic, case-by-case, available time and any other factors. Moreover, during this time students are developing their learning style and may adopt a higher method of learning over time. Nevertheless, such limitations are potentially minimal.

## CONCLUSION

Medical students in a primary health care setting are using multiple educational resources suitable and relevant to primary health care rotation. Further research using intervention methods based on our findings would support enhanced learning in primary healthcare rotation.
